# Clinical Analysis of Y Chromosome Microdeletions and Chromosomal Aberrations in 1596 Male Infertility Patients of the Zhuang Ethnic Group in Guangxi

**DOI:** 10.1007/s43032-024-01568-x

**Published:** 2024-06-05

**Authors:** Mingfang Shi, Shengjun Ma, Li Huang, Chaosheng Huang, Jing Wang, Xuemei Qin, Yibing Luo, Yu Xiong, Ningyu He, Jianghui Zeng

**Affiliations:** 1 Department of Medical Laboratory, The Third Affiliated Hospital of Guangxi Medical University/The Second Nanning People’s Hospital, Nanning, 530031 Guangxi China; 2 Guangxi Key Laboratory of Molecular Immunology Research, Nanning, 530031 Guangxi China; 3 Department of Administrative Office, Nanning Maternity and Child Health Hospital/Nanning Women and Children’s Hospital, Nanning, 530031 Guangxi China; 4 Department of Neurology, The Third Affiliated Hospital of Guangxi Medical University/The Second Nanning People’s Hospital, Nanning, 530031 Guangxi China

**Keywords:** Male infertility, Y chromosome microdeletions, Chromosome, Zhuang ethnicity

## Abstract

The long arm of the Y chromosome (Yq) contains many amplified and palindromic sequences that are prone to self-reorganization during spermatogenesis, and tiny submicroscopic segmental deletions in the proximal Yq are called Y chromosome microdeletions (YCM). A retrospective study was conducted on male infertility patients of Zhuang ethnicity who presented at Reproductive Medical Center of Nanning between January 2015 and May 2023. Seminal fluid was collected for standard examination. YCM were detected by using a combination of multiplex PCR and agarose gel electrophoresis. Preparation of peripheral blood chromosomes and karyotyping of chromosomes was performed. 147 cases (9.22%) of YCM were detected in 1596 male infertility patients of Zhuang ethnicity. Significant difference was found in the detection rate of YCM between the azoospermia group and the oligospermia group (*P* < 0.001). Of all types of YCM, the highest detection rate was AZFc (*n* = 83), followed by AZFb + c (*n* = 28). 264 cases (16.54%) of sex chromosomal aberrations were detected. The most prevalent karyotype was 47, XXY (*n* = 202). The detection rate of sex chromosomal aberrations in azoospermia group was higher than that in severe oligospermia group and oligospermia group, and the differences were significant (*P* < 0.001). 28 cases (1.57%) of autosomal aberrations and 105 cases (6.58%) of chromosomal polymorphism were identified. The current research has some limitations due to the lack of normal men as the control group but suggests that YCM and chromosomal aberrations represent key genetic factors influencing spermatogenesis in infertile males of Zhuang ethnicity in Guangxi.

## Background

The infertility poses a substantial public health challenge, exerting significant social, psychological, and economic impacts. According to the latest report of the World Health Organization, the prevalence of infertility among couples of childbearing age in the world ranges from 12.6% to 17.5%, affecting approximately one-sixth of the global population [1]. Among them, male infertility accounts for 50%[2]. Among male infertility factors, spermatogenesis disorder is the most common, with clinical manifestations of azoospermia and oligoasthenospermia. Approximately 30% of patients with spermatogenic disorders are associated with genetic factors, including sex chromosomal numerical abnormities (such as 47, XXY Klinefelter syndrome (KS)), certain autosomal gene mutations, and Y chromosomal structural or gene aberrations [3]. Consequently, conducting genetic tests related to male reproduction holds substantial importance for guiding clinical treatment, enhancing the effectiveness and safety of assisted reproductive technology (ART), and facilitating preimplantation genetic testing (PGT). The Zhuang ethnicity constitutes the largest minority ethnic group in Guangxi, China. There are few reports on the genetic analysis of male infertility of the Zhuang ethnicity. This study aimed to explore the association between genetic aberrations and the phenotype of male infertility in the Zhuang ethnic group by retrospectively analyzing the results of Y chromosome microdeletions (YCM) and peripheral blood chromosome karyotypes in 1596 male infertility patients with abnormal sperm count from the Zhuang ethnic group in Guangxi.

## Methods

### Patients

1104 male patients with azoospermia, 292 male patients with severe oligospermia, and 200 male patients with oligospermia who received treatment at the Reproductive Medical Center of the Second Nanning People’s Hospital in Guangxi, China between January 2015 to May 2023 were selected. These male infertility patients were the Zhuang ethnic people that is the most populous ethnic minority in Guangxi. The patients’ ages ranged from 22 to 55 years, with an average age of 35 years. The medical ethical committee of the Second Nanning People’s Hospital’s Ethics Committee has given its approval for this study (No. Y2013163). The study was conducted in compliance with the ethical guidelines of the Declaration of Helsinki.

### Semen Analysis

Patients were instructed to abstain from ejaculation for 2 to 7 days. Following patients’ semen samples were procured via masturbation. Semen examination followed the standardized protocols by the "World Health Organization Laboratory Manual for the Examination and Processing of Human Semen (5th Edition)". Azoospermia was defined as the patient whose semen was centrifuged at 3000 g for 15 min and no sperm was found after sedimentation by three centrifugation sedimentation analysis. Severe oligospermia was identified as a sperm concentration of less than 5 × 10^6^·mL^−1^. Oligospermia was classified as 5 × 10^6^·mL^−1^ ≤ sperm concentration < 15 × 10^6^·mL^−1^.

### Identification of Y Chromosome Microdeletions

Genomic DNA was extracted from venous blood treated with EDTA-K_2_ anticoagulant by employing the Labaid 824 Nucleic Acid Extractor through the magnetic bead method. The Y chromosome microdeletions gene detection kit (Shenzhen Yaneng Biotech Co., Ltd.) was utilized for multiplex PCR amplification. This kit specifically amplifies fifteen sequence tag sites (STS) in the AZF region, of which six are defined as partitioned STS, including the AZFa region: sY84, sY86; AZFb area: sY127, sY134; AZFc region: sY254, sY255, eight STS that confirm whether the entire segment is completely missing, including AZFa region: sY82, sY1064, sY1065, sY88; AZFb region: sY105, sY121, sY192, sY153, along with a heterochromatin tag sY160. The sex-determining region (SRY) on the Y chromosome’s short arm was utilized as a sex abnormality control, and the human zinc finger protein gene ZFX/Y was employed as an internal experimental control. All steps were performed according to the reagent manual. The amplified target DNA fragments were analysed by using agarose gel electrophoresis, and the results were observed, photographed, and interpreted by using the Gel Imaging System (Bio-rad, USA).

### Analysis of Peripheral Blood Chromosomal Karyotype

Heparin-anticoagulated peripheral blood was subjected to lymphocyte culture and chromosome preparation. Chromosomal karyotype was examined via the chromosome G-banding technology. At least 20 cells were counted, 5 complete metaphase mitoses were analysed, and any number and structural aberrations observed were recorded. Karyotype analysis and chromosome abnormalities were named according to the International System for Human Cytogenomic Nomenclature (ISCN 2013).

### Statistical Analysis

Data were analysed by using SPSS 24.0 statistical software. Group comparisons were performed by using the chi-square test, and a *P* < 0.05 was considered statistically significant.

## Results

### Overall Situation of Y Chromosome Microdeletions

**Fig. 1:**
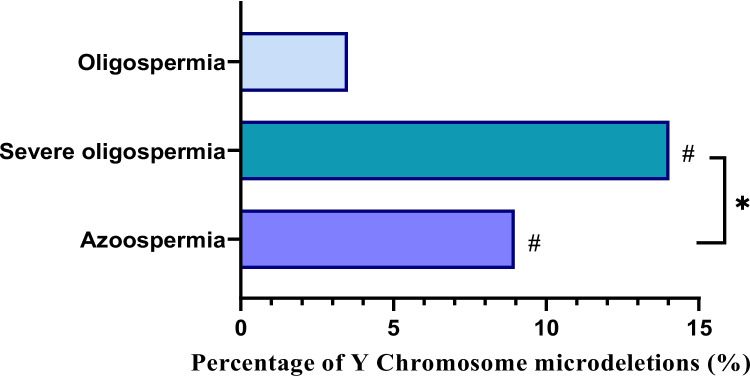
Percentage of Y Chromosome microdeletions (YCM) in azoospermia group, severe oligospermia group and oligospermia group. *Comparison with severe oligospermia group, *P* < 0.05; ^#^Comparison with oligospermia group, *P* < 0.05

Among the 1596 patients with abnormal sperm count from the Guangxi of Zhuang ethnic group, 147 cases (9.22%) exhibited YCM. The rates of YCM in the azoospermia group, severe oligospermia group, and oligospermia group were 8.97% (99/1104), 14.04% (41/292), and 3.50% (7/200), respectively. The highest prevalence of YCM was noted in the severe oligospermia group, significantly differing from the azoospermia group and oligospermia group (*P* = 0.010, *P* < 0.001). The difference between the azoospermia group and oligospermia group was also statistically significant (*P* = 0.009). The results are illustrated in Fig. 1.

### Missing Situation in each Zone of AZF

**Fig. 2:**
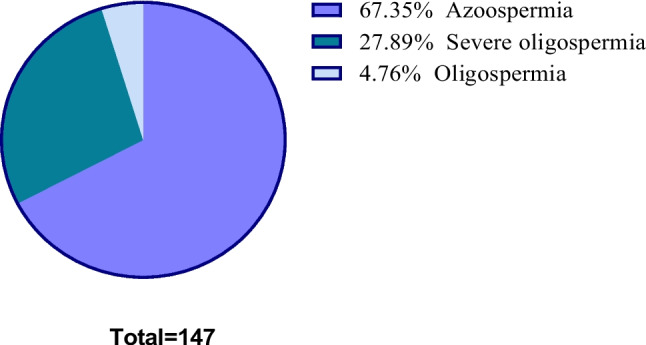
The proportion of patients with YCM in the azoospermia, severe oligozoospermia, and oligozoospermia

In the cohort of 147 patients presenting YCM, the distributions among the azoospermia group, severe oligospermia group, and oligospermia group were 67.35% (99/147), 27.89% (41/147), and 4.76% (7/147) respectively. The proportion of the azoospermia group was the highest, which was significantly higher than that of the severe oligospermia group and oligospermia group, and the differences were statistically significant (*P* < 0.001). The discrepancy was also statistically notable between the severe oligospermia group and the oligospermia group (*P* < 0.001), as show in Fig. 2. Among all types of microdeletions, the highest detection rate was AZFc (83 cases), accounting for 56.46% (83/147), followed by AZFb + c (28 cases), accounting for 19.05% (28/147), which had the highest detection rate in combined deletions. AZFa, AZFb, AZFa + b, and AZFa + b + c accounted for 11 cases, 14 cases, 1 case, and 10 cases, respectively. The results are illustrated in Table 1.

**Table 1:** Percentage of azoospermia factor (AZF) type in azoospermia group, severe oligospermia group and oligospermia group

AZF type	Azoospermia (*n* = 99)	Severe oligospermia (*n* = 42)	Oligospermia (*n* = 7)	Percentage (%)
AZFa	9	0	2	7.48(11/147)
AZFb	11	1	2	9.53(14/147)
AZFc	41	39	3	56.46(83/147)
AZFa + b	1	0	0	0.68(1/147)
AZFb + c	27	1	0	19.05(28/147)
AZFa + b + c	10	0	0	6.80(10/147)
Percentage(%)	67.35(99/147)*#	27.89(41/147)#	4.76(7/147)	100(147/147)

^*^Comparison with severe oligospermia group, *P* < 0.05; ^#^Comparison with oligospermia group, *P* < 0.05

### Chromosomal Karyotype Abnormalities

Among 1596 patients with abnormal sperm count of Zhuang ethnicity in Guangxi, 264 cases (16.54%) were found to have abnormal sex chromosomal karyotypes, including 256 cases (23.19%) in the azoospermia group, 7 cases (2.40%) in the severe oligospermia group and 1 case (0.50%) in the oligospermia group. The highest rate of sex chromosomal karyotype abnormalities was observed in the azoospermia group, and the difference was statistically significant when contrasted with the severe oligospermia group and oligospermia group (*P* < 0.001). However, there was no statistically significant difference between the severe oligospermia group and the oligospermia group (*P* = 0.204). The most common sex chromosomal abnormality identified was the 47, XXY (KS) with a total of 202 cases (12.66%), of which 200 cases were from the azoospermia group and 2 cases from the severe oligospermia group. Other frequently identified sex chromosomal abnormalities included the 46, X, del(Y)(q?), 47, XYY, and 46, XX, with 5, 3, and 3 cases, respectively. The results are shown in Table 2. Autosomal abnormalities were detected in a total of 28 cases (1.57%), including 10 cases of the azoospermia group (0.91%), 16 cases of the severe oligospermia group (5.47%), and 2 cases of the oligospermia group (1.00%). The highest rate of autosomal abnormalities was observed in the severe oligospermia group, which was statistically significantly higher than the rates in both the azoospermia group (*P* < 0.001) and oligospermia groups (*P* = 0.009). However, the difference between the azoospermia group and oligospermia group was not statistically significant (*P* = 1.000). The autosomal abnormal karyotype with the highest detection rate was Robertson translocation 45, XY, der (13; 14) (q10; q10), 3 cases (0.19%) were detected. The results are shown in Table 3.

**Table 2:** Percentage of abnormal karyotype in sex chromosomes

Aberrant Karyotype	Azoospermia (*n* = 1104)	Severe oligospermia (* n* = 292)	Oligospermia (*n* = 200)	Overall Percentage (%)	Percentage of abnormal karyotype (%)
47,XXY	200	2		12.66	76.52
47,XYY	1	1	1	0.19	1.14
47,XXY,1qh +	2			0.13	0.75
47,XXY,inv(9)(p11q13),22pstk +	1			0.06	0.38
47,XXYqh-	1			0.06	0.38
47,XXY,21ps +	1			0.06	0.38
47,XXY[55]/46,XX[5]	1			0.06	0.38
47,XXY[60]/46,XX[9]	1			0.06	0.38
47,XY,i(X)(q10)	1			0.06	0.38
47,XX,inv(Y)(p11q12)	1			0.06	0.38
47,XXY[26]/46,XX[75]	1			0.06	0.38
47,XXY,inv(9)(p12q13)	1			0.06	0.38
48,XXY, + mar	1			0.06	0.38
48,XXYY/47,XXY	1			0.06	0.38
46,X,?del(Y)(q11)[73]/45,X[5]	1			0.06	0.38
46,X,del(Y)(q?)	5			0.31	1.88
46,XY,del(Y)(q?)	2			0.13	0.75
46,XY,del(Y)(q12)	2			0.13	0.75
46,X,t(Y;11)(q11;p15)	1			0.06	0.38
46,XX	3			0.25	1.51
46,XX[50]	1			0.06	0.38
46,XX,21pstk + [50]	1			0.06	0.38
46,XY,t(12;Y)(q13;q12)	1			0.06	0.38
46,XYY,t(9;19)(p12;q12)		1		0.06	0.38
46,X,add(Y)(q?)	1			0.06	0.38
46,X,del(Y)(q?)[80]/45,X[20]	1			0.06	0.38
46,X,del(Y)(q11)	2			0.13	0.75
46,XX[28]/47,XXY[24]	1			0.06	0.38
46,XY(Y = 22)/45,XO	1			0.06	0.38
46,XY[20]	2			0.13	0.75
46,XY,(Y = 22)	1			0.06	0.38
46, XY, Y slightly larger	1			0.06	0.38
46, XY, Y slightly smaller	1			0.06	0.38
46,XY, small Y	1			0.06	0.38
46,Y,t(X;22)(q26;q11.2)	1			0.06	0.38
46,XY,t(1;Y)(q36;q11)		1		0.06	0.38
46,X,-Y, + mar	1			0.06	0.38
46,X, + mar		1		0.06	0.38
45,X[70]/46,XY[30]	1			0.06	0.38
45,X[19]/46,XY[41]	1			0.06	0.38
45,X[35]/46,XYqh-[20]	1			0.06	0.38
45,X[32]/46,X,r(Y)?[26]		1		0.06	0.38
45,X[36]/46,XYqh-[14]	1			0.06	0.38
45,X[44]/46,X,Yqh-[56]	1			0.06	0.38
45,X[48]/46, XY[3]	1			0.06	0.38
45,X,1qh + [77]/46,XY,1qh + [23]	1			0.06	0.38
45,X[58]/46,XY[42]	1			0.06	0.38
45,X[9]/46,XY[42]	1			0.06	0.38
45,X[74]/46,XY[26]	1			0.06	0.38
45,XY,15pstk +	1			0.06	0.38
Percentage (%)	23.19*^#^ (256/1104)	2.40 (7/292)	0.50 (1/200)	16.54 (264/1596)	100.00 (264/264)

^*^Comparison with severe oligospermia group, *P* < 0.05; ^#^Comparison with oligospermia group, *P* < 0.05

**Table 3:** Percentage of abnormal autosomal karyotypes

Abnormal autosomal karyotype	Azoospermia (*n* = 1104)	Severe oligospermia (*n* = 292)	Oligospermia (*n* = 200)	Overall percentage (%)	Percentage of abnormal Karyotype (%)
45,XY,der(13;14)(q10;q10)	1	1	1	0.19	10.70
45,XY,der(21;22)		1		0.06	3.57
45,XY,der(14;21)(q10;q10)		2		0.13	7.14
46,XY,t(3;16;6;5)(q10;q23;q11;q11)		1		0.06	3.57
46,XY,add(15)(p13)	1			0.06	3.57
46,XY,add(22)(p11.2)	1			0.06	3.57
46,XY,inv(1)(q32q21)		1		0.06	3.57
46,XY,inv(1)(q32q244)	1			0.06	3.57
46,XY,t(1;15)(q21;p12)		1		0.06	3.57
46,XY,t(1;19)(q21;p13.3)		1		0.06	3.57
46,XY,t(1;2)(q23;p23)	1			0.06	3.57
46,XY,t(1;5)(q23;q33)			1	0.06	3.57
46,XY,t(11;17)(p15;q21)	1			0.06	3.57
46,XY,t(13;14)(q12;q24)		1		0.06	3.57
46,XY,t(14;15)(p11;q11),21S +		1		0.06	3.57
46,XY,t(4;17)(q35;q23)		1		0.06	3.57
46,XY,t(4;6)(q31;q15)	1			0.06	3.57
46,XY,t(8;21)(q12;p11)	1			0.06	3.57
46,XY,t(1;7)(p34.3;q22)	1			0.06	3.57
46,XY,t(1;14)(q32.2;q24)		1		0.06	3.57
46,XY,t(1;16)(q25;q25.1)		1		0.06	3.57
46,XY,t(2;13)(q21;q12)		1		0.06	3.57
46,XY,t(3;16;6;5)(q10;q23;q11;q11)		1		0.06	3.57
46,XY,inv(9)(p11q12),t(2;13)(q10;q22)		1		0.06	3.57
47,XY, + 21	1			0.06	3.57
Percentage (%)	0.91* (10/1104)	5.47^#^ (16/292)	1.00 (2/200)	1.75 (28/1596)	100.00 (28/28)

^*^Comparison with severe oligospermia group, *P* < 0.05; ^#^Comparison with oligospermia group, *P* < 0.05

### Distribution of Chromosomal Polymorphism

In the 1596 instances of abnormal sperm count among the Zhuang ethnic population of Guangxi, chromosomal polymorphism were identified in 105 instances (6.58%), including 74 cases in the azoospermia group (6.70%), 22 cases in the severe oligospermia group (7.50%), and 9 cases in the oligospermia group (4.50%). The differences were not statistically significant among the three groups (*P* = 0.393). The most frequently detected chromosomal karyotype was 46,X,Yqh-, identified in 23 cases (1.41%), inclusive of 19 cases in the azoospermia group, 3 cases in the severe oligospermia group, and 1 case in the oligospermia group. The findings are depicted in Table 4.

**Table 4:** Percentage of chromosomal polymorphism karyotypes

Polymorphism karyotype	Azoospermia (*n* = 1104)	Severe Oligospermia (*n* = 292)	Oligospermia (*n* = 200)	Overall percentage (%)	Percentage of abnormal karyotype (%)
46,X,Yqh-	19	3	1	1.44	21.90
46,X,Y?qh-	1			0.06	0.95
46,XY,1qh +	11	3	1	0.94	14.29
46,XY,22 ps +	5	1	1	0.44	6.67
46,XY,22pstk +	5	2		0.44	6.67
46,X,Yqh +	6	1		0.44	6.67
46,XY,15 ps +	1	3		0.25	3.81
46,XY,15pstk +	1			0.06	0.95
46,XY,9qh +	1	3		0.25	3.81
46,XY,13pstk +		3	1	0.25	3.81
46,XY,15cenh +	1		1	0.13	1.90
46,XY,17 ps	1		1	0.13	1.90
46,X,Yqh-,22 ps +	1			0.06	0.95
46,X,Yqh-,1qh +	1			0.06	0.95
46,XY,13cenh +	1			0.06	0.95
46,XY,13cenh + × 2,Yqh-	1			0.06	0.95
46,XY,13 ps +	1			0.06	0.95
46,XY,14 ps +	1	1		0.13	1.90
46,XY,14pstk +	1			0.06	0.95
46,XY,21pstk +	3		2	0.31	4.76
46,XY,21pstkstk		1		0.06	0.95
46,XY14pstk + ,21pstk +	1			0.06	0.95
46,XY,inv(9)(p11q12)	2			0.13	1.90
46,XY,inv(9)(p11q13)	7	1	1	0.56	8.57
46,XY,inv(9)(p12q13)	2			0.13	1.90
Percentage (%)	6.70 (74/1104)	7.50 (22/292)	4.50 (9/200)	6.58 (105/1596)	100.00 (105/105)

### Analysis of Chromosomal Aberrations in Conjunction with Y Chromosome Microdeletions

In the 1596 instances of abnormal sperm count of Zhuang ethnicity in Guangxi, 37 cases (2.32%) of chromosomal karyotype abnormalities or chromosome polymorphisms in combination with Y chromosome microdeletions were detected. The most frequently detected combination was sex chromosomal abnormalities with YCM (24 cases), followed by chromosomal polymorphism with YCM (11 cases), and the least were autosomal abnormalities with YCM (2 cases). Among 37 cases of chromosomal karyotype abnormalities or chromosome polymorphisms with YCM, 35 cases were azoospermia patients, and only 2 cases were severe oligospermia patients. Additionally, the highest detected type of YCM was AZFb + c (24 cases), followed by AZFc (6 cases), AZFa + b + c (5 cases), and AZFa (2 cases). The results are shown in Table 5.

**Table 5:** The number and clinical manifestations of patients with chromosomal abnormalities in conjunction with Y chromosome microdeletions

Types of chromosomal abnormalities	Chromosomal karyotype	Frequency	AZF type	Clinical manifestations
Sex chromosomal abnormalities (*n* = 24)	45,X[58]/46,XY[42]	1	AZFc	azoospermia
	45,X[44]/46,X,Yqh-[56]	1	AZFb + c	azoospermia
	45,X[36]/46,XYqh-[14]	1	AZFb + c	azoospermia
	45,X[48]/46, XY[3]	1	AZFb + c	azoospermia
	45,X[74]/46,XY[26]	1	AZFb + c	azoospermia
	45,X[35]/46,XYqh-[20]	1	AZFb + c	azoospermia
	46,XY,del(Y)(q?)	1	AZFc	azoospermia
	46,XY,del(Y)(q?)	3	AZFb + c	azoospermia
	46,XY,del(Y)(q?)	1	AZFa + b + c	azoospermia
	46,XY,del(Y)(q12)	1	AZFc	azoospermia
	46,XY,del(Y)(q12)	1	AZFb + c	azoospermia
	46,X,-Y, + mar	1	AZFb + c	azoospermia
	46,XX	1	AZFa + b + c	azoospermia
	46,XX,21pstk + [50]	1	AZFa + b + c	azoospermia
	46,XY,(Y = 22)	1	AZFb + c	azoospermia
	46,X,del(Y)(q?)[80]/45,X[20]	1	AZFb + c	azoospermia
	46,X,del(Y)(q11)	1	AZFa + b + c	azoospermia
	46,XX[50]	1	AZFa + b + c	azoospermia
	46,XY(Y = 22)/45,XO	1	AZFb + c	azoospermia
	46,XY, small Y	1	AZFb + c	azoospermia
	46,X,?del(Y)(q11)[73]/45,X[5]	1	AZFb + c	azoospermia
	48,XXYY/47,XXY	1	AZFa	azoospermia
Autosomal abnormalities (*n* = 2)	46,XY,add(22)(p11.2)	1	AZFa	azoospermia
	46,XY,t(1;2)(q23;p23)	1	AZFb + c	azoospermia
Chromosomal polymorphism (*n* = 11)	46,XY,1qh +	1	AZFb	Severe oligospermia
	46,X,Yqh-	1	AZFc	Severe oligospermia
	46,X,Yqh-	1	AZFc	azoospermia
	46,X,Yqh-	6	AZFb + c	azoospermia
	46,XY,15 ps +	1	AZFc	azoospermia
	46,X,Yqh-,1qh +	1	AZFb + c	azoospermia
Total		37		

## Discussion

The Y chromosome contains genes that are essential for testicular maturation and the initiation and sustenance of spermatogenesis in adulthood. Yq encompasses numerous palindromic and amplified sequences, which makes it easy to self-recombination during spermatogenesis. This can engender intra-chromosomal deletions, leading to discrepancies in the copy number of genes on the Y chromosome, thereby contributing to male infertility[4]. YCM is one of the prominent causes of spermatogenic failure, therefore YCM screening has become a routine diagnostic work for infertile men [5, 6].

In this study, the detection rate of YCM in 1596 male infertility patients of Zhuang ethnicity was 9.22%, which was lower than the incidence rate of YCM in male infertility patients reported by Liu et al. in China (16.9%) and the rate of YCM in male infertility patients reported by India (16.1%) [7, 8]. Nonetheless, this is congruent with the 9.2% deletion rate of Y chromosome reported by Wang et al. among infertile males in northeastern China[9]. Prior research has demonstrated significant racial and geographic variation in the detection rate of YCM among infertile men. The rates of YCM range from 12.0% in the US and 24.2% in Iran to a mere 2.0% in nations such as Germany and Austria[10]. The variation of YCM frequency may be caused by various factors such as fluctuations of sample size, bias in subject selection, differences in sequence sites and primers of STS, racial or ethnic differences, and environmental factors. In our study, the incidence of YCM in the azoospermia group and severe oligospermia group was 8.97% and 14.04% respectively, significantly higher than that in the oligospermia group (3.5%), indicating a statistically significant difference. Out of all YCM, 67.35% of the patients with azoospermia and 27.89% of the patients with severe oligospermia were significantly higher than those with oligospermia. The differences among three groups were statistically significant. These data suggest that YCM is salient genetic factor influencing spermatogenesis. The study found that among patients suffering from azoospermatism and oligospermatism, YCM accounted for 3% ~ 29%, rendering it the second most prevalent genetic factor after KS[11]. A meta-analysis of over 10,000 cases revealed that YCM was present in 5% of severe oligospermia patients (sperm count ≤ 1 × 10^6^ /ml) from North America, whereas the incidence in males with normal semen parameters was less than 1%. Consequently, male infertility guidelines in North America and Europe recommend YCM testing was only detected for men with sperm concentrations of ≤ 1 × 10^6^/ml [12]. Our research is consistent with these studies, confirming a close correlation between YCM and spermatogenic abnormalities.

In couples with recurrent pregnancy loss (RPL), male partners with abnormal semen parameters were significantly more probable to have a Y deletion (37.5%, 3/8) than men with normal semen parameters (19.6%, 10/51) and fertile controls (FC) without abnormal semen parameters (0/20); YCMs were presented in 13 male partners (32.5%) of 40 couples with RPL, while the incidence of YCM in fertile controls with no abnormal semen parameters was 0 ( 0/20) [13]. Similarly, another study [14] showed a higher prevalence of YCM in couples with the presence of RPL (16%, 7/43) compared to fertile controls (0/43). A systematic analysis of YCM and assisted reproductive technology (ART) pregnancy outcomes showed [15] a significant decrease in fertilization rate in the YCM group compared to the normal group. Some males with defective YCM and severe oligozoospermia or azoospermia are able to reproduce by ART. However, fathers may transmission microdeletions to sons produced by intracytoplasmic sperm injection (ICSI) and confers adverse effects on male fertility [16]. Although YCM are present in 25-55% of men with extreme testicular pathology (e.g., hypospermatogenesis, sperm maturation arrest) and in 5–25% of men with severe oligozoospermia or azoospermia [17], the Y deletion should be viewed as a cause of oligozoospermia/azoospermia rather than as a direct cause of "infertility". Nevertheless, in clinical practice, the screening of YCM is still very useful in helping clinicians to identify the etiology of male infertility and determine reasonable management strategies for patients.

The gene housed on the long arm of the Y chromosome, which governs spermatogenesis, is collectively known as the azoospermatism factor (AZF). In 1996, Vogot et al. classified AZF into three distinct regions: AZFa, AZFb, and AZFc, each of which corresponds to different stages of spermatogenesis [11]. In our study, the detection rate of AZFc in YCM was the highest, which was consistent with the literature reports [18–20], suggesting that deletions within the AZFc region hold significant relevance to semen quality. Some studies suggest that there is a certain relationship between AZF deletion type and abnormal sperm count in patients with different pathological types [21]. The deletion of the AZFa region typically leads to Sertoli cell-only syndrome (SCOS), and since genes within the AZFa sites are expressed prenatally in germ cells, absence of these genes may instigate developmental apoptosis of germ cells, resulting in SCOS [22, 23]. Consequently, the diagnosis of complete absence of the AZF a region means that it is almost impossible to obtain testicular sperm for ICSI. The absence of AZFb region is characterized by spermatogenesis arrest, and spermatogenesis is blocked at the stage of spermatocyte, so spermatogoniums and primary spermatocytes are still visible in the testis, but there is no spermatogenesis. Therefore, for azoospermia patients with complete deletion of AZFb (including AZFb + c deletion), artificial insemination by a donor (AID) is recommended [24, 25]. Patients presenting with AZFc deletion may exhibit normal sperm count, oligospermatism, or azoospermatism. Moreover, this condition is inheritable in male offspring. Patients with AZFc deficiency can have sperm extracted from testicular tissue and have children via ICSI, avoiding unnecessary surgical treatment [26, 27]. Our study not only detected the deletion of AZFc, but also found types of deletions such as AZFa, AZFb, AZFb + c, AZFa + b, and AZFa + b + c, indicating that deletions in various regions of AZF may occur in male infertility patients. Clinically, the detection outcomes of different AZF regions can offer reference data for genetic counseling, clinical diagnosis, therapeutic strategy, and the selection of assisted reproductive technology, thereby mitigating unnecessary medicinal and surgical treatment.

The chromosomal count, structural anomalies, and polymorphism are paramount genetic factors contributing to male infertility [28]. In this study, the incidence of sex chromosomal abnormalities was 16.54% in 1596 men with abnormal sperm count of Zhuang ethnicity, which was considerably higher than the incidence of autosomal abnormalities (1.75%) and chromosomal polymorphism (6.58%). Moreover, it surpasses the reports in national and international literature [29, 30]. The incidence of sex chromosomal abnormalities in the azoospermia group was 23.19%, which was significantly higher than that in the severe oligospermia group (2.40%) and the oligospermia group (0.50%), and the differences were statistically significant. However, no significant difference was noted in the rates of autosomal abnormalities and chromosomal polymorphism between the azoospermia group and the oligospermia group. These data suggest that, sex chromosomal abnormalities are the principal genetic factors causing male spermatogenic disorders among chromosomal abnormalities.

Klinefelter Syndrome (KS), also known as congenital testicular underdevelopment or primary microorchidism, is a condition where male possess additional X chromosome. It is the most common sex chromosomal abnormalities. Data show that the incidence rate of non chimeric KS in newborn boys is 1/660 [31], the prevalence rate in infertile men is about 3% ~ 4%, and that in azoospermia patients is as high as 10% ~ 12% [31, 32]. Males with KS typically present with small testicles, low testosterone levels, elevated gonadotropin levels, and some may exhibit abnormal genital organs, including cryptorchidism, hypospadias, micropenis, etc.[33]. Research indicates that most patients with KS undergo normal development during puberty, but their testicular volume seldom exceeds 4-5 mL, and secondary sexual characteristics appear 3–4 years late on average [34].

The abnormal phenotypes of KS patients primarily originate from the dosage effect of escaping inactive genes on the extra X chromosome, which interferes with the normal development process, including the development of the reproductive system in fetal period and adolescence, as well as other possible symptoms [35]. In our study, 202 cases of KS with a karyotype of 47, XXY were detected, accounting for the highest percentage of sex chromosomal abnormalities (76.52%). The phenotype was mainly azoospermatism, indicating that KS affected the normal development of males reproduction, mainly manifested as spermatogenesis disorders, which was consistent with previous literature reports [32, 33]. In the past, it was widely believed that patients with KS were infertile and had no possibility of fatherhood. However, advances in assisted reproductive technology have made it possible for men with KS to have offspring through testicular sperm extraction technique (TESE) and ICSI in recent years [36]. Patients with KS are easily overlooked and missed due to their lack of obvious clinical symptoms before puberty. Most adult patients with KS seek medical help due to fertility issues. In patients presenting with small testicles and azoospermatism, further laboratory tests such as chromosomal analysis and testicular biopsy are required to rule out or initially screen for infertility caused by KS. Early accurate diagnosis, intervention, and treatment of KS are essential to select the appropriate ART for patients as soon as possible.

In this study, six cases of Robertsonian translocation were detected, accounting for 0.38% of autosomal abnormalities. The main manifestation of Robertsonian translocation was severe oligospermia. Previous research have also found that some male Rob (13q14q) carriers present with azoospermatism or severe oligospermatism, and chromosome 13 and 14 variations were significantly related to male testicular spermatogenesis or fertility [37]. Robertson translocation means that two proximal centromere chromosomes (group D, G) are broken near the centromere respectively and then reconnected, usually retaining the entire long arm of the two chromosomes but lacking only two short arms. Because of the small short arm, the few inherited genes and the unclear genetic effect, so the phenotype and intellectual development of the carriers with Robertsonian translocation are generally normal. However, the gamete (ova or sperm) of Robertsonian translocation carriers will show an imbalance of chromosome nondisjunction during meiosis, thus there is a risk of giving birth to the offspring of unbalanced translocation. A large number of gene duplications or deletions can have serious genetic effects, such as recurrent abortion, adverse pregnancy history and infertility in Robertsonian translocation carriers, and these carriers are mostly detected when they give birth to children with abnormal chromosome [38]. For patients diagnosed with recurrent miscarriage, poor pregnancy history, infertility, it is crucial to conduct peripheral blood chromosome examination as early as possible to exclude the influence of chromosomal abnormalities. If Robertsonian translocation is indicated, appropriate fertility counseling should be provided, such as considering the use of ART and PGT [39]. These measures aim to alleviate the financial and emotional physical burdens placed on the patient as well as physical trauma.

Chromosome polymorphism, also known as normal chromosomal variation, refers to some constant small chromosomal variations observed in healthy individuals, including differences in the size of chromosomal fragments or chromosomal bands. Initially, studies postulated that these differences typically do not induce genetic effects nor manifest as significant pathologies, given their high frequency, they were categorized as a form of polymorphism. But through over a decade of research, more and more scholars believe that chromosomal polymorphism can cause genetic effects such as reproductive abnormalities [40, 41]. In our investigation involving 1596 infertile males from the Zhuang ethnic group, the detection rate of chromosome polymorphism is 6.58%, which is significantly lower than the 34.5% incidence reported by Penna-Videau et al. in 84 cases of male infertility [42], and the 8.7% incidence reported by Mau in 150 cases of male infertility [43]. In addition, no significant differences were noted in the incidence of chromosome polymorphism among azoospermia group, severe oligospermia group and oligospermia group in our study. These data suggest that chromosomal polymorphism may exert a lesser influence on spermatogenesis in the Zhuang ethnic group. At the same time, we found that 46, X, Yqh - had the highest detection rate in chromosome polymorphism, accounting for 1.44% of all infertile people, and accounting for 21.90% of chromosome polymorphism, and most of them were azoospermia patients, which was inconsistent with the report by Penna Videau et al. [42] that Yqh + was stated to be common chromosome polymorphism in male infertility.

Research conducted by Kayhan Yakin et al. [44] found that the chromosome polymorphism of male infertile patients was higher than that of normal men. They believed that the increase of heterochromatin polymorphism in infertile men seemed not only accidental, but also could not be considered as a normal variation. Polymorphic heterochromatin may have harmful effects on the genetic composition of sperm, and more attention should be paid to infertile men with heterochromatin polymorphism [44]. Our study presents some limitations as we did not incorporate a control group of males with normal sperm count, thus we are unable to definitively ascertain that chromosome polymorphism is not associated with male infertility, pending further accumulation and investigation of clinical data. In a study, though, chromosome analysis and YCM analysis of the parents of the 35 individuals displaying chromosomal polymorphism probands with chromosomal polymorphisms, the results revealed that 30 patients acquired the polymorphisms from their parents; the karyotype of the parents is similar to that of their respective probands, but there is no similar history of adverse reproduction. YCM analysis of the fathers of YCM patients showed that none of the YCM was inherited from the paternal side. Thus, the infertility of the probands was suggested to be a result of YCM, and not polymorphisms in the Y chromosome [45].

In our investigation, a total of 37 cases of chromosome abnormality combined with YCM were detected, among which sex chromosome abnormality combined with YCM was the most common (24 cases), followed by chromosome polymorphism combined with YCM (11 cases), suggesting that when screening the genetic causes of male infertility patients, YCM detection and chromosome karyotype analysis should be performed simultaneously to avoid missed detections. This is conducive to early diagnosis of the cause, early treatment and selection of appropriate assisted reproductive technology for pregnancy. Among the 37 patients with concurrent chromosomal abnormalities and YCM, 35 cases were identified as azoospermia patients, suggesting that the chromosome abnormality with YCM significantly impact male fertility. Consequently, even in cases of chromosomal polymorphism, the importance of conducting YCM detection cannot be underestimated. The current research has some limitations due to the lack of normal men as the control group but suggests that YCM and chromosomal aberrations represent key genetic factors influencing spermatogenesis in infertile males of Zhuang ethnicity in Guangxi.

In any case, although studies have suggested that YCM and chromosomal abnormalities are associated with sub-/non-fertile men, due to the lack of data from the normal men having normal sperm parameters in this study, it seems more appropriate to consider YCM as a cause of oligozoospermia/azozoospermia rather than as a direct cause of "infertility". Some studies have also indicated that there is no significant relationship between abnormal sperm parameters (including count, morphology and motility) and the risk of RPL [46, 47]. However, our results have shown that YCM and chromosomal abnormalities are important indices that should not be ignored. And in clinical practice, the screening of YCM and chromosomal abnormalities will help clinicians to identify the etiology of male infertility and determine reasonable management strategies for patients.

## Conclusions

Male infertility within the Zhuang ethnic group of Guangxi exhibits strong correlations with Y-chromosome microdeletions and chromosomal abnormalities. It is evident that these genetic elements represent the primary causes of spermatogenic disorders in males of Zhuang ethnic group. Therefore, it is crucial to perform an examination for Y-chromosome microdeletions in conjunction with routine chromosomal karyotype analysis in patients presenting with male infertility. Joint assessments aid clinicians in further elucidating the underlying disease etiology, determining the need for assisted reproduction, and assessing the necessity for pre-implantation diagnosis.

## Data Availability

All data generated or analysed during this study are included in this published article.

## Abbreviations

AID: Artificial insemination by a donor
ART: Assisted reproductive technology
AZF: Azoospermia factor
ICSI: Intracytoplasmic sperm injection
KS: Klinefelter syndrome
PGT: Preimplantation genetic testing
RPL: Recurrent pregnancy loss
SCOS: Sertoli cell-only syndrome
SRY: Sex-determining region
STS: Sequence tag sites
TESE: Testicular sperm extraction technique
YCM: Y chromosome microdeletions
AID: Artificial insemination by a donor
